# Resveratrol Co-Treatment Attenuates the Effects of HIV Protease Inhibitors on Rat Body Weight and Enhances Cardiac Mitochondrial Respiration

**DOI:** 10.1371/journal.pone.0170344

**Published:** 2017-01-20

**Authors:** Burger Symington, Rudo F. Mapanga, Gavin R. Norton, M. Faadiel Essop

**Affiliations:** 1 Cardio-Metabolic Research Group (CMRG), Department of Physiological Sciences, Stellenbosch University, Stellenbosch, South Africa; 2 Cardiovascular Pathophysiology and Genomics Research Unit, School of Physiology, Faculty of Health Sciences, University of the Witwatersrand, Johannesburg, South Africa; University of PECS Medical School, HUNGARY

## Abstract

Since the early 1990s human immunodeficiency virus (HIV)/acquired immunodeficiency syndrome (AIDS) emerged as a global health pandemic, with sub-Saharan Africa the hardest hit. While the successful roll-out of antiretroviral (ARV) therapy provided significant relief to HIV-positive individuals, such treatment can also elicit damaging side-effects. Here especially HIV protease inhibitors (PIs) are implicated in the onset of cardio-metabolic complications such as type-2 diabetes and coronary heart disease. As there is a paucity of data regarding suitable co-treatments within this context, this preclinical study investigated whether resveratrol (RSV), aspirin (ASP) or vitamin C (VitC) co-treatment is able to blunt side-effects in a rat model of chronic PI exposure (Lopinavir/Ritonavir treatment for 4 months). Body weights and weight gain, blood metabolite levels (total cholesterol, HDL, LDL, triglycerides), echocardiography and cardiac mitochondrial respiration were assessed in PI-treated rats ± various co-treatments. Our data reveal that PI treatment significantly lowered body weight and cardiac respiratory function while no significant changes were found for heart function and blood metabolite levels. Moreover, all co-treatments ameliorated the PI-induced decrease in body weight after 4 months of PI treatment, while RSV co-treatment enhanced cardiac mitochondrial respiratory capacity in PI-treated rats. This pilot study therefore provides novel hypotheses regarding RSV co-treatment that should be further assessed in greater detail.

## Introduction

The human immunodeficiency virus (HIV) has become a global pandemic over the last 30 years, affecting ~34 million people (2010), of which 2.7 million were children under the age of 15 years old [1]. Although it began as a relatively insignificant disease during the 1980s, HIV prevalence has since escalated to become one of the leading, global causes of morbidity and mortality [2,3]. For example, the World Health Organization [4] reported that by the end of 2015 there were 36.9 million HIV-positive individuals globally, while 1.2 million deaths occurred as a result of HIV-related causes. Of note, sub-Saharan Africa is burdened with highest number of HIV-positive persons and this places considerable strain on health-care facilities and also poses a strong challenge to sustained economic growth and development in this region [5].

The initiation of combination antiretroviral (ARV) treatment in 1996 revolutionized HIV treatment and has since stunted HIV prevalence and lowered viral load [6]. Thus the life expectancy of HIV-positive individuals has robustly increased since the introduction of ARVs. However, the trajectory of HIV-related morbidity has since begun to shift, i.e. primarily from opportunistic infections and immune dysfunction to associated cardio-metabolic diseases [7]. In support, the incidence of cardiovascular complications in this population group has begun to increase [8], usually manifesting relatively late during disease progression [8,9]. However, it remains unclear whether such effects are due to the virus itself and/or ARV treatment [7].

HIV infection is a risk factor for cardiovascular diseases (CVD) development and this can occur by direct effects of viral proteins on the heart and vasculature [10,11], thereby contributing to the onset of a pro-atherogenic state [12,13]. ARV treatment is also linked to the onset of cardio-metabolic complications [8,9], with protease inhibitors (PIs) and nucleoside reverse transcriptase inhibitors strongly implicated in this process [8,9]. In particular, studies indicate that PIs pose a substantial threat as prolonged treatment can trigger damaging, downstream intracellular effects on the cardiovascular system [14,15].

Although the mechanisms of PI-mediated cardio-metabolic side-effects are not entirely clear, higher oxidative stress and mitochondrial dysfunction emerge as major culprits mediating this process [14,16–18]. In addition, HIV itself may elicit similar effects [17]. For example, chronic inflammation in the vasculature is a risk factor and predictor for CVD onset [19] as it can trigger a pro-atherogenic state [20], thereby increasing the risk for myocardial infarction and/or strokes [20]. Of note, HIV PI-induced dyslipidemia and inflammation are two major risk factors for the development of cardiovascular complications in HIV patients. PIs can induce inflammation by various mechanisms that include endoplasmic reticulum stress, an accumulation of intracellular free cholesterol and lipids (thereby activating the unfolded protein response in hepatocytes and macrophages), increasing the release of inflammatory cytokines, and promoting foam cell formation and apoptosis in macrophages [21–23]. Together these studies demonstrate that PIs and HIV itself can perturb intracellular metabolism and signaling pathways in the cardiovascular system that subsequently contribute to the development of cardio-metabolic complications, thereby affecting the overall well-being of HIV-positive persons receiving ARVs.

However, despite progress made to understand the underlying mechanisms driving HIV- and/or ARV-mediated onset of cardio-metabolic complications, there is a paucity of data regarding the most suitable therapeutic strategies to combat this growing problem. In light of this, the current preclinical study evaluated three well-known therapeutic agents (resveratrol [RSV] and vitamin C [VitC][anti-oxidants], aspirin [ASP] [anti-inflammatory]) in a rat model of chronic PI exposure (Lopinavir/Ritonavir treatment for 4 months). Here our rationale was to select compounds that are readily available (‘‘off the shelf”) and priced at a reasonable cost if considered for large scale usage by HIV-positive individuals. Our findings show that RSV in particular elicited beneficial outcomes by reversing PI-mediated weight changes and also enhancing cardiac mitochondrial respiratory function. This study therefore provides novel hypotheses regarding RSV co-treatment that should be further evaluated in targeted experiments.

## Materials and Methods

### Animals

All animals were treated in accordance with the Guide for the Care and Use of Laboratory Animals of the National Academy of Sciences (NIH publication No. 85–23, revised 1996) and performed with the approval of the Animal Ethics Committee of Stellenbosch University (Stellenbosch, South Africa). Male Wistar rats were acquired at 150–180 grams body weight and were housed in standard-sized IVC rat cages (3 animals per cage), subjected to 12:12 h light-dark cycles with *ad libitum* access to standard chow and water.

### Drug treatments

Rats were randomly divided into 6 experimental groups (n = 6 per group): a) control, b) vehicle control (jelly), c) PI treatment, d) PI + RSV, e) PI + ASP and f) PI + VitC. Treatment dosages were calculated using body surface area based on the following formula [24]:

$$Human\;Equivalent\;Dose\;\left(\frac{mg}{kg}\right)=Animal\;dose\;\left(\frac{mg}{kg}\right)\times \frac{Animal\;Km}{Human\;Km}$$

The HIV PI (Alluvia™—Neelsie Pharmacy, Stellenbosch, South Africa) containing 200 mg Lopinavir/ 50 mg Ritonavir per tablet was employed for this study. For the RSV (Terraternal, Santa Clara CA), ASP (Disprin™ tablets, Reckitt &Benckiser Healthcare, Hull, UK) and VitC (purchased in the form of powdered sodium ascorbate—Enhance, Compli-Med, South Africa) co-treatments we employed dosages of 200, 300 and 300 mg per kilogram body weight, respectively, as previously described [25–29]. Raspberry flavored gelatin (Tower Jelly, Bokomo Foods, Bellville, South Africa) was combined with pure gelatin (ratio of 4:1) as a vehicle for treatment regimens except for the control that received standard rat chow. Jelly blocks were prepared by mixing flavored and pure gelatin in a pre-determined amount of boiling water and stirred until completely dissolved. The jelly solution was then directly pippeted into ice trays with volumes ranging from 1.5–2.5 ml, depending on rat weights. Each of the various drug regimens (PI, PI + RSV, PI + ASP, PI + VitC) was added to different wells of the ice tray once the jelly solution had cooled, and trays were subsequently placed at 4°C until it had set. Jelly blocks without any drug treatments were employed as an additional control group in our study (vehicle control). Thus we administered jelly blocks to rats for the vehicle control, PI and PI + treatment groups. However, the control group did not receive any jelly blocks. Treatments were administered daily (between 16:00–18:00; to coincide with the start of the dark cycle) for a 4-month period.

### Blood collection and organ weight determination

After 4 months the rats were fasted overnight, sedated (2% isoflurane) and 1 ml of blood was drawn from the jugular vein. Plasma was isolated using standard procedures as described before [16] and collected samples analyzed by the National Health Laboratory Services (Tygerberg, Western Cape, South Africa) to assess cholesterol (total, HDL, LDL) and triglyceride levels. In order to determine whether the various treatments induced any gross anatomical changes, body weight and weight gain were determined together with weights for harvested organs (heart, right and left ventricles, liver, total and retroperitoneal fat)—normalized to tibial length. The total fat in this case refers to the sum of the two retroperitoneal fat pads. For weight changes, the rats were weighed every third day.

### Echocardiography

At the end of the 4-month period, rats were sedated as described earlier (refer previous section). Two-dimensional directed M-mode echocardiography (Acuson Cypress echocardiograph with a 7.0 MHz transducer; Siemens, Germany) was performed by an experienced investigator (GRN) who was blinded to the different experimental groups, in the short axis of the left ventricle from a parsternal long axis view as previously described [30]. Left ventricular wall thickness and diameter values were obtained using the leading edge method to identify points of reference on the endocardial surface of the septral and posterior walls [30]. We determined left ventricular end-diastolic diameter (LVEDD), left ventricular end-systolic diameter (LVESD), left ventricular end-systolic posterior wall thickness (LVESPWT), and left ventricular end-diastolic posterior wall thickness (LVEDPWT). We also assessed the duration from end diastole of one beat to end diastole of the subsequent beat in order to calulate heart rate. Left ventricular endocardial fractional shortening (FSend), an index of systolic chamber function, was calculated as: end-diastolic diameter minus end-systolic diameter/ end-diastolic diameter; expressed as a percentage. Echocardiography could not be performed at the beginning of the study (baseline) as rats were too small to obtain accurate M-mode images. As we are unable to obtain apical 4-chamber views of the heart which incorporate a clear endocardial border around the whole circumference of the left ventricle, we we did not assess systolic chamber function by calculating ejection fraction using the biplane Simpson approach. We also did not employ the Teichholz method of calculationg ejection fraction from M-mode images as this approach is highly senistive to inaccuracies produced by differences in remodeling.

### Isolation of mitochondria

After sedation (5% isoflurane), rats were culled by exsanguination and hearts quickly removed and mitochondria isolated as described before [31]. Here tissues were transferred into a small beaker containing ice-cold Buffer A (100 mM KCl, 50 mM MOPS, 5.0 mM MgSO_4_, 1.0 mM EGTA and 1.0 mM ATP). Hearts were trimmed of atria, connective tissue and fat and then washed once with 5 ml Buffer A. Ventricular tissues were thereafter chopped thoroughly into small pieces with a dissection scissors using Buffer B (Buffer A + 2 mg/ml BSA -fatty-acid free). After rinsing, chopped heart pieces were placed in 15 ml Buffer B and homogenized with a polytron homogenizer (Ultra-Turrax, Staufen, Germany) for three sec. Homogenized hearts were subsequently centrifuged at 580 x *g* at 4°C for 7 min where after the supernatant was filtered into a clean centrifugation tube. The remaining pellet was resuspended in 7.5 ml Buffer B and centrifuged as before; thereafter the filtered supernatant was added to the supernatant collected previously. This was followed by centrifugation at 580 x *g* at 4°C for 7 min to rinse, where after the remaining supernatant was then centrifuged at 3, 000 x *g* for 7 min at 4°C. Subsequently, the supernatant was discarded and the pellet containing sub-sarcolemmal mitochondria was resuspended in 0.3 ml KME buffer (100 mM KCl, 50 mM MOPS, 0.5 mM EGTA, pH 7.4).

### Evaluation of mitochondrial respiration

Respiratory rates were polarographically measured using a Clark-type electrode (Hansatech Instruments, London, UK) at 37°C with constant stirring as previously described before by us with modifications [32]. Here, mitochondrial oxygen consumption was assessed in isolated sub-sarcolemmal mitochondria (0.25 mg mitochondrial protein/mL) in respiration buffer containing 100 mM KCl, 50 mM MOPS, 5 mM KH_2_PO_4_, 1 mM EGTA, and 1 mg/mL BSA. State 3 and 4 respiration were measured with glutamate + malate (10 and 5 mM, respectively); malate + pyruvate (5 mM and 10 mM, respectively); and malate + carnitine palmitoyl (5 mM and 20 mM, respectively). Mitochondrial oxygen uptake during state 3 respiration was measured after the addition of 10 mM ADP to maximally stimulate respiration. State 4 respiration was determined by measuring mitochondrial oxygen uptake upon complete phosphorylation of ADP to ATP. The ADP/O ratio, a measure of mitochondrial oxidative phosphorylation efficiency, was calculated as the ratio between the ADP added and oxygen consumed during ADP phosphorylation. The rate of ADP phosphorylation was calculated as nanomoles of ADP phosphorylated per minute during state 3 respiration as described before [33].

Mitochondria were considered viable where the respiratory control ratio (RCR) (state 3/state 4) were ≥4. The RCR and the ADP/O ratios were calculated according to Estabrook, 1967 [34]. All mitochondrial polarographic studies were normalized to total mitochondrial protein content, determined using the Bradford assay [35].

### Statistical analysis

All data are expressed as mean ± SEM and values considered significant when p < 0.05. Statistical analysis was done in a very focused manner to assess our hypotheses. Here we compared specific groups, i.e. a) control (vehicle) versus PI and b) PI vs. each respective co-treatment using the Student’s t-test (Graph Pad prism v5. San Diego CA).

## Results

### Morphologic changes

In order to determine whether the various treatments induced any gross anatomical changes, body weight and weight gain were determined together with weights for harvested organs—normalized to tibial length. Table 1 shows that after the 4 months of treatment, PI-treated rats showed a significant decrease in body weight vs. the vehicle control group (S1 Data). However, co-treatment with all three interventions significantly attenuated the PI-induced decrease in body weight, with especially RSV and VITC showing a robust weight gain. In parallel, weights for both ventricles and the liver were significantly lower but this difference disappeared after correcting for tibia length (S2 Data). For RSV and VITC treatments, this was accompanied by increased fat weight gain. There were additional significant changes but only by a minor percentage to be considered functionally significant.

**Table 1:** Morphometric Changes following 4 Months of Treatment.

	Control	Vehicle Control	PI	PI + RSV	PI + ASP	PI + VitC
**Body Weight**	363.5 ± 5.4	371.4 ± 5.0	340.8 ± 2.4^###^	360.4 ± 3.4^@@@^	348 ± 2.5^$ $ $^	369.8 ± 3.8***
**Weight Gained**	179.5 ± 2.8	186 ± 2.9	140.1 ± 2.4^###^	161.2 ± 2.7^@@@^	148.7 ± 2.7	169.5 ± 2.6***
**Tibia Length**	42.08 ± 0.5	42.83 ± 0.26	41 ± 0.41^##^	42.58 ± 0.69	42.63 ± 0.62^$^	42.88 ± 0.4**
**Heart Weight/Tibia Length**	0.02 ± 0.001	0.02 ± 0.001	0.02 ± 0.001	0.02 ± 0.001	0.02 ± 0.001	0.02 ± 0.001
**Left Ventricular(LV) weight**	0.72 ± 0.04	0.75 ± 0.03	0.64 ± 0.03^#^	0.66 ± 0.06	0.61 ± 0.03^##^	0.66 ± 0.03^#^
**LV Weight/Tibia Length**	0.017 ± 0.0007	0.017 ± 0.0007	0.016 ± 0.0009	0.016 ± 0.0013	0.015 ± 0.0007^#^	0.015 ± 0.0007
**Right Ventricular(RV) weight**	0.19 ± 0.01	0.17 ± 0.01	0.15 ± 0.01^#^	0.15 ± 0.01	0.13 ± 0.01	0.15 ± 0.0
**RV Weight/Tibia Length**	0.004 ± 0.0003	0.004 ± 0.0003	0.004 ± 0.0002	0.004 ± 0.0002	0.003 ± 0.0002	0.004 ± 0.0002
**Liver Weight**	11.83 ± 0.64	11.86 ± 0.49	10.35 ± 0.46^#^	12.01 ± 0.72	11.82 ± 0.6	12.23 ± 0.35**
**Liver Weight/Tibia Length**	0.28 ± 0.01	0.28 ± 0.01	0.25 ± 0.01	0.28 ± 0.02	0.28 ± 0.01	0.29 ± 0.01*
**Retroperitoneal Fat Pad weight**	5.03 ± 0.89	5.67 ± 0.83	3.81 ± 0.48	6.64 ± 1.04^@^	5.00 ± 0.65	6.28 ± 0.82*
**RP Fat Weight/Tibia Length**	0.12 ± 0.02	0.13 ± 0.02	0.09 ± 0.01	0.16 ± 0.03^@^	0.12 ± 0.02	0.15 ± 0.02*
**Testicular Fat Pad weight**	8.42 ± 1.05	8.42 ± 0.49	6.01 ± 0.36^##^	9.01 ± 1.14^@^	7.27 ± 1.03	8.9 ± 0.9*
**T Fat Weight/Tibia Length**	0.2 ± 0.02	0.19 ± 0.01	0.15 ± 0.01^##^	0.22 ± 0.03^@^	0.17 ± 0.02	0.21 ± 0.02*

#p<0.05 PI vs. vehicle control

## p<0.01 vs. vehicle control

### p<0.001 PI vs. vehicle control

@ p<0.05 PI vs. PI + RSV

@@@ p<0.01 PI vs. PI + RSV

$ p<0.05 PI vs. PI + ASP

$$$ p<0.001 PI vs. PI + ASP

* p<0.05 PI vs. PI + VitC

** p<0.01 PI vs. PI + VitC

*** p<0.001 PI vs. PI + VitC.

### Metabolic parameters 4 months of treatment

Blood TG, total cholesterol and HDL levels remained consistent for all groups after 4 months of PI treatment. However, PI-treated rats receiving RSV and VitC exhibited an increase in circulating LDL blood levels vs. vehicle controls (p < 0.05) (Table 2, S3 Data).

**Table 2:** Blood Metabolite Levels following 4 Months of Treatment.

	Control	Vehicle Control	PI	PI + RSV	PI + ASP	PI + VitC
TG	0.9 ± 0.1	0.9 ± 0.2	1.3 ± 0.2	1.1 ± 0.2	1.6 ± 0.2	1.1 ± 0.1
Chol	2.1 ± 0.09	2.2 ± 0.14	2.1 ± 0.18	2.1 ± 0.14	2.2 ± 0.16	2.3 ± 0.01
HDL	0.6 ± 0.05	0.6 ± 0.03	0.6 ± 0.05	0.5 ± 0.04	0.6 ± 0.05	0.6 ± 0.02
LDL	1.1 ± 0.1	1.2 ± 0.2	0.9 ± 0.1	1.1 ± 0.0^$^	0.9 ± 0.1	1.2 ± 0.1*

$ *p* < 0.05 PI vs. PI + ASP

* *p* < 0.05 PI vs. PI + VitC.

### Analysis of endocardial fractional shortening (FSend) with the use of echocardiography

To establish the effects of each treatment on systolic function, various left ventricular dimensions were assessed by echocardiography. These results were used to calculate FSend, an index of systolic chamber function, at the end of the 4-month treatment period. No significant changes were found for left ventricular systolic function (Table 3, S4 Data).

**Table 3:** Left Ventricular Parameters as Measured with Echocardiography after 4 Months of Treatment.

	Control	Vehicle Control	PI	PI + RSV	PI + ASP	PI+VitC
**LVEDD (mm)**	6.7 ± 0.13	6.6 ± 0.16	6.4 ± 0.16	6.8 ± 0.14	7.1 ± 0.09	6.8 ± 0.11
**LVESD (mm)**	3.6 ± 0.12	3.5 ± 0.15	3.8 ± 0.14	3.8 ± 0.14	3.9 ± 0.13	3.8 ± 0.15
**LVEDPWT (mm)**	2.1 ± 0.07	2.0 ± 0.09	1.9 ± 0.04	1.9 ± 0.05	2.0 ± 0.07	2.0 ± 0.06
**LVESPWT (mm)**	2.6 ± 0.1	2.6 ± 0.09	2.4 ± 0.09	2.6 ± 0.08	2.6 ± 0.08	2.5 ± 0.07
**R-R Interval (sec)**	0.17 ± 0.004	0.18 ± 0.005	0.18 ± 0.003	0.17 ± 0.004	0.18 ± 0.004	0.18 ± 0.003
**% FSend**	45.9 ± 1.6	47.8 ± 1.8	40.5 ± 2.8	44.4 ± 0.9	44.5 ± 1.7	43.4 ± 3.0

All results expressed as mean ± SEM (n = 6). LVEDD – Left Ventricular End Diastolic Diameter; LVESD – Left Ventricular End Systolic Diameter; LVESPWT – Left Ventricular End Systolic Posterior Wall Thickness; LVEDPWT – Left Ventricular End Diastolic Posterior Wall Thickness; FSend – left ventricular endocardial fractional shortening.

### Analysis of mitochondrial respiration

Our results on mitochondrial oxygen consumption indicate that State 3 respiration was not significantly altered with PI treatment compared to the vehicle control (although values were generally lower) (Table 4, S5 Data). The substrate combinations of pyruvate + malate and glutamate and malate activate dehydrogenases with the reduction of nicotinamide adenine dinucleotide (NADH) then feeding electrons into Complex I and down the thermodynamic cascade through the Q-cycle and Complex III of the electron transport system to Complex IV and O_2_. Here RSV treatment significantly enhanced State 3 respiration compared to sole PI treatment. However, ASP and VitC treatments resulted in a significantly lower ADP: O and RCR, respectively. We also assessed fatty acid-mediated respiration by employing palmitoyl–carnitine + malate as substrates but no significant effects were found in this instance.

**Table 4:** Function of Isolated Left Ventricular Mitochondria after 4 Months of PI Treatment.

	Control	Vehicle Control	PI	PI + RSV	PI + ASP	PI + VitC
**Glutamate + Malate State III**	200 ± 12	175 ± 24	150 ± 13	154 ± 10	196 ± 19	180 ± 22
**Glutamate + Malate State IV**	86 ± 12	71 ± 6	64 ± 5	65 ± 6	71 ± 10	72 ± 8
**Glutamate + Malate RCR**	2.53 ± 0.28	2.51 ± 0.21	2.37 ± 0.22	2.43 ± 0.2	2.9 ± 0.26	2.52 ± 0.21
**Glutamate + Malate ADP: 0**	2.0 ± 0.1	2.2 ± 0.4	2.7 ± 0.2	2.7 ± 0.2	1.9 ± 0.2^$^	2.4 ± 0.3
**Malate + Pyruvate State III**	151 ± 19	164 ± 19	146 ± 11	194 ± 17^@^	161 ± 15	137 ± 11
**Malate + Pyruvate State IV**	67 ± 1	55 ± 4	63 ± 8	68 ± 3	58 ± 4	59 ± 5
**Malate + Pyruvate State RCR**	2.4 ± 0.32	2.96 ± 0.2	3.2 ± 0.3	2.93 ± 0.3	2.80 ± 0.17	2.37 ± 0.15*
**Malate + Pyruvate State ADP: O**	2.78 ± 0.31	2.61 ± 0.30	2.81 ± .025	2.15 ± 0.22	2.59 ± 0.25	3.0 ± 0.23
**Palmitoyl – Carnitine + Malate State III**	178 ± 31	203 ± 37	172 ± 17	141 ± 7	184 ± 27	162.7 ± 19
**Palmitoyl – Carnitine + Malate State IV**	90 ± 4	72 ± 10	83 ± 9	66 ± 5	71 ± 10	69 ± 11
**Palmitoyl – Carnitine + Malate RCR**	1.96 ± 0.3	3.14 ± 0.51	2.2 ± 0.34	2.2 ± 0.24	2.71 ± 0.37	2.58 ± 0.5
**Palmitoyl – Carnitine + Malate ADP: O**	2.50 ± 0.39	2.3 ± 0.4	2.43 ± 0.21	2.9 ± 0.14	2.37 ± 0.28	2.61 ± 0.33

All results expressed as mean ± SEM (n = 6). @ p<0.05 PI vs. PI + RSV; $ p<0.05 PI vs. PI + ASP; * p<0.05 PI vs. PI + VitC

RCR – respiratory control ratios; ADP: O – adenosine phosphate: oxygen.

## Discussion

Previous studies demonstrated the occurrence of various metabolic changes in HIV-infected patients (reviewed by [7] and that ARVs (particularly PIs) can trigger a number of metabolic alterations (e.g. hyperlipidemia, lipodystrophy, insulin resistance) that constitute key parameters of the metabolic syndrome [8,9]. Such perturbations are linked to increased development of premature atherosclerosis and to a higher risk for CVD onset in HIV-positive individuals. Although it remains unclear whether such effects occur due to the HIV infection itself and/or ARV treatment, there is a need to assess suitable co-treatments to ultimately improve clinical management and well-being of HIV-positive persons on long-term ARV treatment. Considering this, the current pilot study evaluated three well-known therapeutic interventions (RSV, ASP and VitC) in rats receiving PI treatment for a period of four months. Here our data reveal that PI-treated rats displayed significantly lowered body weights compared to matched controls. However, all co-treatments significantly increased body weight gain after four months of treatment. This was accompanied by significant increases in fat weight gain in PI-treated rats. Levels of various blood metabolite levels did not show wide-scale differences except that LDL levels were moderately increased with both RSV and VitC co-treatments. In addition, *in vivo* cardiac function and isolated mitochondrial respiratory capacity remained unaltered after four months of PI administration. However, RSV co-treatment significantly increased State 3 respiration compared to PI-treated rats.

### Co-treatments attenuate PI-mediated decrease in body weight

After four months, PI-treated groups displayed the lowest body weight/weight gain when compared to the other treatment groups. Similar findings were obtained following PI treatment of mice [36,37] and also within the clinical setting, e.g. the Study of Fat Redistribution and Metabolic Change in HIV Infection (FRAM) demonstrated lipoatrophy in HIV-infected men [38]. Lipodystrophy in Highly Active Antiretroviral Therapy (HAART)-treated HIV-positive individuals presents clinically as decreased peripheral adipose tissue and increased visceral/central adipose tissue [39]. For our study, there was a trend that showed PI treatment attenuated retroperitoneal fat pad mass compared to the other groups. This correlates with results from others who found significantly reduced retroperitoneal fat pad mass in mice treated with Ritonavir/Lopinavir [36,37]. It has been suggested that this may be linked to PI-mediated lowering of dietary intake in rodents [36,37], although this would need to be assessed in our model to determine whether indeed the case. It is therefore likely that other mechanisms may be responsible for such effects, e.g. PI treatment can attenuate adipokine levels (adiponectin, leptin) [40] and subsequently increase overall energy expenditure thereby contributing to weight loss [41,42]. By contrast, previous work from our laboratory found that two months of PI treatment resulted in a significant increase in rat body weight [16]. The precise explanation for such discordant data remains unclear although likely reasons include length of PI treatment (two versus four months) and also the mode of drug delivery (mini-osmotic pumps versus jelly blocks). The current study also established that all three co-treatments—especially RSV and VitC—attenuated PI-mediated effects on body weight/weight gain. We are unclear regarding the underlying mechanisms responsible and whether PIs reduces or if the co-treatments increase fat content. Further studies are required to answer this intriguing question.

### Blood metabolite profile and cardiac function not altered with PI treatments

For the current study we found no profound changes in blood profiles (total cholesterol, HDL, triglycerides) of rats treated with PIs ± co-treatments. However, RSV and VitC co-treatments did result in a significant increase in circulating LDL levels. Overall these findings were surprising as HIV infection and ARV treatment are strongly linked to CVD onset that may (in part) occur due to a pro-atherogenic lipid profile. By contrast, our previous preclinical study reported higher circulating LDL levels in rats following two months of PI treatment [16]. In support, it is a relatively common occurrence for ARV-treated individuals (especially on PI regimens) to develop a pro-atherogenic lipid profile [7,43] that typically entails decreased HDL together with increased LDL and triglyceride levels [43,44]. There are several reasons why such changes may not have occurred in our model: a) the HIV infection itself plays a significant role in the development of a pro-atherogenic blood profile, b) the PI steady-state concentration was not high enough to elicit an altered blood profile and c) protocol differences and length of PI treatments may vary between reported studies. What about the co-treatment effects? We are unclear regarding the precise mechanisms but suggest that as both RSV and VitC increased weight gain and fat mass that this may occur as a result of these changes.

As the rats did not present with a pro-atherogenic blood profile we exclude it as an early mediator of direct PI-induced effects on the cardiovascular system in our setting. Likewise, we did not find any significant differences for heart function as evaluated by echocardiographic analysis. In this regard we could not assess baseline cardiac function as rats were too small to obtain accurate M-mode images. Hence we were unable to report on change in values between groups. However, systolic cardiac function at the end of the study was similar between groups. Therefore, any differences in the change in function from baseline to final values between groups are unlikely to be of clinical significance. Moreover, because we could not obtain ideal apical 4-chamber views of the left ventricle (which allows for the assessment of systolic chamber function using both short and long axis views and which is expressed as ejection fraction), systolic chamber function was obtained from M-mode images in the short-axis plane of the left ventricle only (endocardial fractional shortening). Hence we may have failed to detect more subtle changes in systolic function in only the long axis of the heart. However, to-date innumerable studies have demonstrated that in the presence of myocardial pathology, endocardial fractional shortening is sufficient to detect systolic chamber dysfunction. Since the start of the ARV era, there has been an increased incidence of echocardiographic abnormalities and cardiovascular complications observed among HIV patients [45]. For example, Meng et al. (2002) found that PI-containing ARV regimens are associated with left ventricular hypertrophy and diastolic dysfunction [46]. Moreover, HIV-positive children receiving ARVs exhibited relatively high incidences of mild ventricular dysfunction together with progressive increases in left ventricular weight [47]. In addition, we previously found that two months of PI treatment resulted in attenuated *ex vivo* heart function at baseline and in response to ischemia-reperfusion [48]. The exact mechanisms of such PI-induced structural and functional abnormalities are elusive, but hypertension, vascular inflammation, endothelial dysfunction, myocardial calcium handling and impaired protein recycling are all proposed as potential mediators [16,45]. By contrast, the current study suggests that *in vivo* systolic function is unaltered following PI treatment, although it remains to be ascertained whether such treated rat hearts will be more susceptible to stress, e.g. a myocardial infarction insult.

### RSV treatment enhances mitochondrial respiratory function

To gain greater insight into PI-mediated changes we also evaluated mitochondrial respiratory function following four months of treatment. Here PI-treated rat hearts did not display any significant differences in State 3 respiration compared to matched controls. However, VitC and ASP treatments decreased RCR and ADP: O using malate + pyruvate and glutamate + malate, respectively. Although the other parameters remained unaltered this may indicate either a) early mitochondrial dysregulation with such co-treatments, or b) some form of normalization of respiration to control levels. Additional experiments with more sophisticated techniques are required to gain greater insight into this question. By contrast, RSV treatment significantly enhanced mitochondrial respiration (malate + pyruvate). This is consistent with previous work demonstrating that RSV potently induces mitochondrial capacity by activating NAD-dependent deacetylase sirtuin-1 (SIRT1) and peroxisome proliferator-activated receptor gamma coactivator 1-apha (PGC-1α) as downstream targets [49]. Here the effects of RSV were linked to the upregulation of genes for oxidative phosphorylation and mitochondrial biogenesis. Thus further studies are recommended to assess whether this indeed the case in our model of long-term PI administration ± RSV treatment.

### Limitations

As with any study, there are always certain shortcomings that should be noted. Firstly, this research should be regarded as a pilot study aiming to generate sufficient impetus to further assess the compounds here tested (RSV in particular) in improved preclinical models and with greater experimental numbers. Moreover, the findings generated cannot be extrapolated to the clinical setting at this early stage.

Finally, this experimental model did not lead to weight gain and wide-scale alterations in blood metabolite levels as would be expected. As this is a unique experimental model, we speculate that the 4-month time point is too early to elicit such changes and that it is likely that this would be observed at a later time point(s). Thus also raises the point which preclinical model would be most suited to simulate the clinical context. As we have previously employed an osmotic pump mode of delivery that resulted in greater pathology [16,42], we propose that this may be a model better suited to employ for such studies. However, it should be noted that the vehicle (1% ethanol) used to dissolve the PIs may potentially cause problems for longer-term studies and also that this experimental model requires sub-cutaneous implantation of mini-osmotic pumps (to be changed at regular intervals).

## Conclusion

The current pilot study shows that four months of PI treatment impairs body weight gain with no significant changes to cardiac and mitochondrial respiratory function. Here RSV co-treatment attenuated the PI-mediated decrease in body weight and also enhanced cardiac mitochondrial respiratory capacity. This pilot study therefore generates interesting hypotheses regarding RSV and PI co-treatment that should be more comprehensively evaluated.

## Supporting Information

S1 Data Rat Body Weight Data. (XLSX)

S2 Data Organ Weight Data. (XLSX)

S3 Data Blood Metabolite Data. (XLS)

S4 Data Echocardiography Data. (XLSX)

S5 Data Mitochondrial Respiration Data. (XLSX)
